# Coexistence of pulmonary lymphangioleiomyomatosis and pulmonary angiomyolipoma

**DOI:** 10.1186/s12890-016-0286-1

**Published:** 2016-08-15

**Authors:** Xuefeng Sun, Ruie Feng, Ye Zhang, Juhong Shi, Kai-Feng Xu

**Affiliations:** 1 Department of Respiratory Medicine, Peking Union Medical College Hospital, Beijing, 100730 China; 2 Department of Pathology, Peking Union Medical College Hospital, Beijing, China; 3 Department of Thoracic Surgery, Peking Union Medical College Hospital, Beijing, China

**Keywords:** Case report, Lymphangioleiomyomatosis, Angiomyolipoma, Lung

## Abstract

**Background:**

Lymphangioleiomyomatosis (LAM) and angiomyolipoma are two different, but related rare diseases. To the best of our knowledge, pulmonary LAM and pulmonary angiomyolipoma have not previously been observed in the same patient.

**Case presentation:**

A 38-year-old woman presented with a dry cough and left flank pain. She had a right nephrectomy for renal angiomyolipoma 17 years ago. A magnetic resonance imaging scan demonstrated a round mass in the left kidney. A chest computed tomography scan demonstrated scattered small thin-walled cysts and multifocal round nodules in both lungs. A lung biopsy via video-assisted thoracoscopic surgery revealed that the cysts and nodules were manifestations of LAM and angiomyolipomas, respectively. After sirolimus therapy, the renal angiomyolipoma and metastasized pulmonary angiomyolipomas shrank, but pulmonary cysts were unchanged.

**Conclusions:**

LAM and angiomyolipoma are significantly associated, and may coexist in the lungs in rare cases. Sirolimus is effective for both renal angiomyolipoma and metastasized pulmonary angiomyolipomas.

## Background

Lymphangioleiomyomatosis (LAM) is a rare, progressive disease that typically results in cystic lung destruction and predominantly affects women [1]. Angiomyolipoma is a mesenchymal tumor and is the most common benign tumor of the kidney. LAM and angiomyolipoma are different, but substantially related conditions. Both are perivascular epithelial cell tumors and can arise from a common source [2]. Renal angiomyolipoma is present in almost all cases of tuberous sclerosis complex (TSC)-LAM and about half of cases of sporadic LAM. However, to our knowledge, pulmonary LAM and pulmonary angiomyolipoma have not previously been observed simultaneously in the same patient. Here we present the first case of coexisting pulmonary LAM and pulmonary angiomyolipomas.

## Case presentation

A 38-year-old woman presented with a dry cough and left flank pain. Her medical history revealed a right nephrectomy for renal tumor at the age of 21, but she recovered from surgery without complications.

During physical examination, the patient had normal vital signs, with a temperature of 36 °C, pulse of 80 beats/min, BP of 135/80 mmHg, respiratory rate of 18 breaths/min, and oxygen saturation of 98 % on ambient air. She was conscious, with normal cognition. Physical examinations were unremarkable, except for a surgical cicatrix at the right lumber region. Both lungs were clear to auscultation. The abdominal examination was normal, and no percussion tenderness in the region of the kidney was noted.

**Fig. 1:**
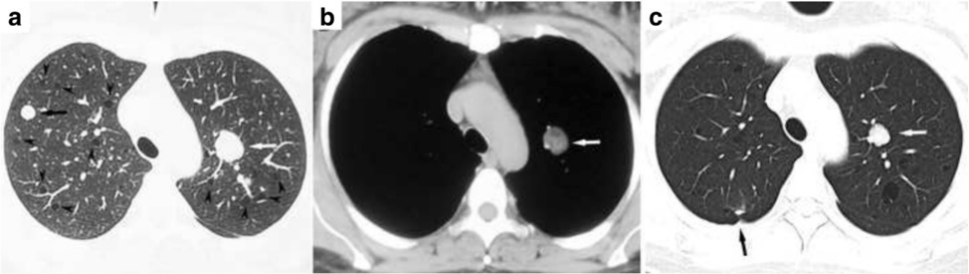
**a** Chest computed tomography scan shows scattered thin-walled cysts (arrowheads) and round nodules (arrows) in the lungs. The largest nodule (2.4 cm in diameter) was located in left upper lobe (white arrow). **b** The pulmonary nodule (white arrow) appears heterogeneous with fatty components on the mediastinal window. **c** A repeat CT 8 months after sirolimus therapy reveals that the diameter of the largest nodule (white arrow) has shrunk to 1.4 cm in diameter. A surgical scar (black arrow) is seen in the right upper lobe

**Fig. 2:**
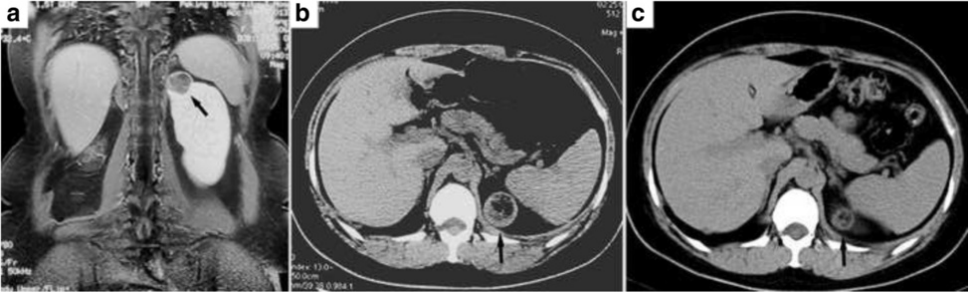
**a** An abdominal magnetic resonance imaging scan and **b** an abdominal CT scan showing a round heterogeneous mass of 4 cm in diameter (arrow) on the upper pole of the left kidney. **c** Eight months after sirolimus therapy, the CT reveals that the mass has shrunk to 2.1 cm in diameter

**Fig. 3:**
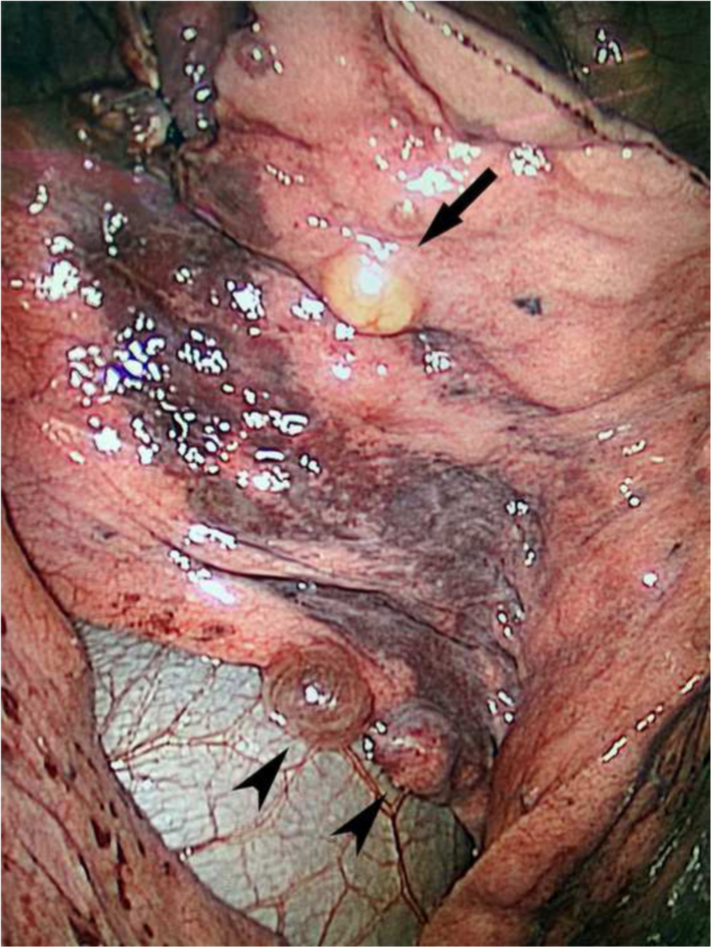
A yellowish nodule (*arrow*) and two cysts (*arrowheads*) are seen on the surface of the lung during video-assisted thoracoscopic surgery

**Fig. 4:**
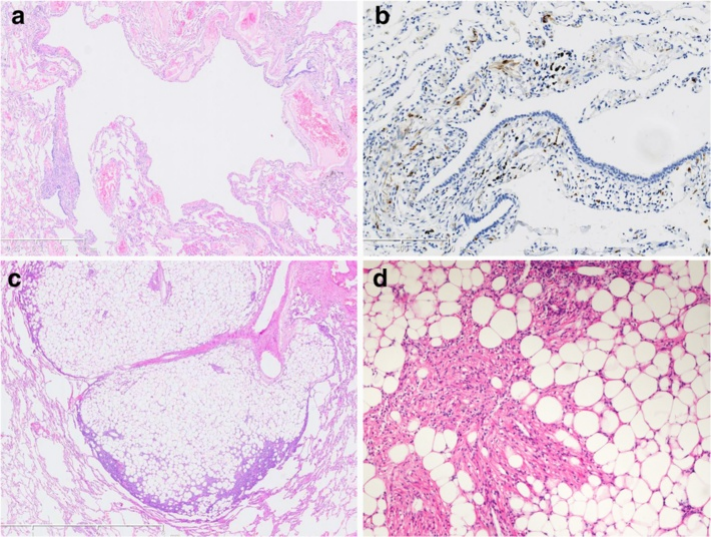
Biopsy specimen of the lung. **a** and **b** The cystic lesion’s walls exhibit the accumulation of some spindle-shaped cells which are positive for HMB45 staining. HE × 10 (**a**), HMB45 × 60 (**b**). **c** and **d** The nodular lesion is a mixture of adipose tissue, smooth muscle, and small blood vessels. HE × 10 (**c**), HE × 60 (**d**)

Complete blood count, urinalysis, renal and hepatic functions were all in normal ranges. Serum VEGF-D was 615 ug/mL (<800 pg/mL). A chest computed tomography (CT) scan demonstrated scattered small thin-walled cysts and 15 round nodules ranging in size from 5 mm to 24 mm in both lungs (Fig. 1a, b). Pulmonary function tests revealed normal ventilation, lung volume and diffusion capacity (FEV1 3.15 L/106.2 %, FEV1/FVC 77.6 %, TLC 112.9 %, and DLCO 91.9 %). Abdominal magnetic resonance imaging scan and CT scan demonstrated a round mass with a diameter of 4 cm on the upper pole of the left kidney (Fig. 2a, b). Histopathological review of the tumor in the right kidney, resected 17 years ago, revealed it to be an angiomyolipoma. Video-assisted thoracoscopic surgery was undertaken, during which cysts and yellowish nodules were seen on the surface of the lung (Fig. 3). Histopathologic analysis of the lung specimen revealed that the cysts coincided with LAM, while the nodules were angiomyolipomas, consistent with that in the right kidney (Fig. 4).

Germinal TSC gene mutation was not detected in this patient; therefore, the pulmonary LAM was considered to be sporadic. Angiomyolipomas in the lungs and the left kidney were predicted to have metastasized from the resected right kidney. After the patient was prescribed 2 mg/d sirolimus, the cough and left flank pain were gradually relieved. A CT scan 8 months later revealed that the mass in the left kidney and some pulmonary nodules had shrunk, but the cysts in both lungs were unchanged (Figs. 1c and 2c).

## Discussion

Pulmonary LAM is a rare lung disease that mainly affects young women of childbearing age [3]. Pathologically, LAM is characterized by the appearance of interstitial collections of atypical smooth muscle cells and cyst formation in the lungs. LAM can be sporadic or associated with TSC. Diffuse, small, symmetrical, thin-walled cysts are characteristic hallmarks of LAM on chest CTs, as was seen in this patient. Occasionally, multifocal micronodular pneumocyte hyperplasia (MMPH) is seen in patients with TSC or LAM on chest CT as random small pulmonary nodules with ground-glass opacity, ranging in size from 1 mm to 1 cm [4, 5]. MMPH nodules are composed of thickened fibrotic alveolar septa lined by pleomorphic type II pneumocytes. In our case, pulmonary nodules were initially thought to be an unusual manifestation of MMPH. However, these nodules were consolidated and much larger than those described in the literature, which motivated the decision to perform a biopsy.

Angiomyolipoma is a rare mesenchymal tumor composed of adipose tissue, smooth muscle cells and thick-walled vessels. It is the most common benign tumor of the kidney. Both LAM and angiomyolipoma belong to a family of tumors collectively termed neoplasms with perivascular epithelioid differentiation, which are now referred to as perivascular epithelial cell tumors (PEComas) [2]. HMB-45 and melan-A are present in angiomyolipoma, differentiating it from other primary and secondary mesenchymal or primary epithelial tumors [6]. In our case, HMB-45 and melan-A were both expressed in both renal and pulmonary tumors, confirming the diagnosis of angiomyolipoma. Most angiomyolipomas occur in the kidney; in rare cases, extrarenal angiomyolipoma may arise from the liver, mediastinum, uterus, spermatic cord, retroperitoneum, spleen, skin and lung [7]. Renal angiomyolipomas may occur in association with TSC, pulmonary LAM or occur as a sporadic finding. TSC has been estimated to be present in approximately 10 % of cases of clinically diagnosed renal angiomyolipomas [8].

Both LAM and angiomyolipoma are closely related to TSC, and appear to arise from a common source [9]. Furthermore, pulmonary LAM and renal angiomyolipoma are also closely related. Renal angiomyolipomas have been reported to occur in up to 100 % of patients with TSC-LAM, and in up to 50 % of those with sporadic LAM [10–13].

Angiomyolipoma is usually benign, but occasionally metastasizes distally. Both sporadic pulmonary angiomyolipoma, and pulmonary metastases originating from renal angiomyolipoma have been described since 1995 [14]; and in a few cases pulmonary metastasis occurred years after the nephrectomy for renal angiomyolipoma [7, 15]. In this case, pulmonary and left renal angiomyolipomas occurred simultaneously 17 years after nephrectomy for right renal angiomyolipoma, and pulmonary and renal angiomyolipoma histopathology was consistent. Therefore, distal metastases seem the most likely explanation. Regrettably, molecular analysis (e.g. p53 gene mutation) [16, 17], which could better chatacterize the homology of renal and pulmonary angiomyolipomas, was not performed in this patient.

Both pulmonary LAM and renal angiomyolipoma are rare diseases; and pulmonary angiomyolipoma has been described in only a few cases. Although the coexistence of LAM and renal angiomyolipoma is noted in about 50 % of sporadic LAM patients, to the best of our knowledge, coexistence of pulmonary LAM and pulmonary angiomyolipoma has not previously been described. Genetic analysis suggested that LAM cells could originate from the smooth muscle cells of renal angiomyolipoma, and be transported through the circulation [18]. Therefore, one explanation for the pulmonary coexistence is that the renal angiomyolipoma released tumor cells to the lung through the blood flow during resection [15], allowing both metastatic pulmonary angiomyolipomas and LAM to form over a long period of time. Another explanation is that the patient might already have had LAM when she underwent nephrectomy since the patient’s pulmonary evaluation 17 years ago was performed via chest X-ray, and this technique is not sufficiently sensitive to detect an early LAM. In this case pulmonary angiomyolipomas may have metastasized from the kidney later. Whichever hypothesis is correct, LAM and angiomyolipoma appear to be related.

The mTOR inhibitor, sirolimus, stabilizes lung function and improves some measures of quality of life in patients with LAM [19, 20]. Sirolimus has been approved by the US Food and Drug Administration for the treatment of moderate-to-severe pulmonary LAM. Sirolimus is also efficacious in controlling tumor volume in patients with renal angiomyolipomas [21]. Everolimus, a derivative of sirolimus, also represents a promising treatment for LAM and renal angiomyolipoma [22, 23]. Surgery or embolism is an alternative choice when an intervention is indicated in patients with renal angiomyolipomas [24]. Sirolimus therapy was chosen in this patient in consideration of the coexistence of pulmonary LAM, pulmonary angiomyolipomas and renal angiomyolipoma. The outcome in this case demonstrated that sirolimus was effective not only for renal angiomyolipoma, but also for metastasized pulmonary angiomyolipomas.

## Conclusions

LAM and angiomyolipoma are substantially related, and may coexist in the lungs in rare cases. Sirolimus can effectively resolve both renal angiomyolipoma and metastasized pulmonary angiomyolipomas.

## Abbreviations

LAM: Lymphangioleiomyomatosis
MMPH: Multifocal Micronodular Pneumocyte Hyperplasia
PEComas: Perivascular Epithelial Cell Tumors
TSC: Tuberous Sclerosis Complex
